# Managing the Sick Child in the Era of Declining Malaria Transmission: Development of ALMANACH, an Electronic Algorithm for Appropriate Use of Antimicrobials

**DOI:** 10.1371/journal.pone.0127674

**Published:** 2015-07-10

**Authors:** Clotilde Rambaud-Althaus, Amani Flexson Shao, Judith Kahama-Maro, Blaise Genton, Valérie d’Acremont

**Affiliations:** 1 Epidemiology and Public Health department, Swiss Tropical and Public Health Institute, Basel, Switzerland; 2 University of Basel, Basel, Switzerland; 3 National Institute for Medical Research, Tukuyu Medical Research Center, Tukuyu, Tanzania; 4 City Medical Office of Health, Dar es Salaam City Council, Dar es Salaam, Tanzania; 5 Infectious Disease Service, University Hospital, Lausanne, Switzerland; 6 Department of Ambulatory Care and Community Medicine, University Hospital, Lausanne, Switzerland; Kenya Medical Research Institute - Wellcome Trust Research Programme, KENYA

## Abstract

**Objective:**

To review the available knowledge on epidemiology and diagnoses of acute infections in children aged 2 to 59 months in primary care setting and develop an electronic algorithm for the Integrated Management of Childhood Illness to reach optimal clinical outcome and rational use of medicines.

**Methods:**

A structured literature review in Medline, Embase and the Cochrane Database of Systematic Review (CDRS) looked for available estimations of diseases prevalence in outpatients aged 2-59 months, and for available evidence on i) accuracy of clinical predictors, and ii) performance of point-of-care tests for targeted diseases. A new algorithm for the management of childhood illness (ALMANACH) was designed based on evidence retrieved and results of a study on etiologies of fever in Tanzanian children outpatients.

**Findings:**

The major changes in ALMANACH compared to IMCI (2008 version) are the following: i) assessment of 10 danger signs, ii) classification of non-severe children into febrile and non-febrile illness, the latter receiving no antibiotics, iii) classification of pneumonia based on a respiratory rate threshold of 50 assessed twice for febrile children 12-59 months; iv) malaria rapid diagnostic test performed for all febrile children. In the absence of identified source of fever at the end of the assessment, v) urine dipstick performed for febrile children <2years to consider urinary tract infection, vi) classification of ‘possible typhoid’ for febrile children >2 years with abdominal tenderness; and lastly vii) classification of ‘likely viral infection’ in case of negative results.

**Conclusion:**

This smartphone-run algorithm based on new evidence and two point-of-care tests should improve the quality of care of <5 year children and lead to more rational use of antimicrobials.

## Introduction

The rapid spread of resistant pathogens worldwide calls for urgent action to improve the rational use of antimicrobials. In low and middle income countries, where infectious diseases childhood mortality is high[1], substandard drugs, auto-medication and health workers (HWs)’ over-prescription of antimicrobials are driving the rapid spread of antimicrobial resistance. [2,3] Recent experience in malaria case management has shown that using appropriate diagnostic tools (malaria rapid diagnostic tests—mRDT) has the potential to improve rational use of antimalarial[4–6] without negative impact on health outcome. [4,7–10] Unfortunately it has often been accompanied with an increased antibiotics prescription[4,5,11], reflecting the challenge faced by HWs in front of a negative malaria test result, where diagnostic tools and skills to rule out bacterial diseases are scarce.

To support HWs’ decision making in the management of a sick child in low resource settings, WHO and UNICEF have developed the Integrated Management of Childhood Illness (IMCI) clinical algorithm in the mid 90’s[12]. The IMCI guidelines rely on the classification of patients based on clinical signs that can be recognized by trained HWs even if their educational background is limited[12]; no laboratory test was included in the IMCI version of 2008: presumptive malaria treatment was recommended for all febrile children (in high malaria risks area). Other causes of fever were not considered (except if the child presented also a complaint leading to another branch of the algorithm). With the advent of new evidence on etiologies and management of childhood illness and reliable point-of-care tests (POCTs), there is a need to rethink the IMCI guidelines and to propose a new algorithm for the management of acute medical illness for children aged 2 to 59 months living in low resource settings. This new algorithm should integrate reliable POCTs and, when the latter are not available, clinical predictors for acute illnesses, so that guidance based on best available evidence is provided to clinicians to decide on withholding antimalarials and antibiotics when not beneficial to the child. When no tool or strong evidence is available to propose appropriate procedures, expert opinion should be sought.

An algorithm developed for HWs in remote primary health care facilities (PHCF) should rely on simple clinical signs and easy-to-perform POCTs. Its structure should remain simple, although addressing a larger set of diseases may require a more complex one. The use of hand-held electronic technology to deliver the algorithm may facilitate the use of a complex clinical algorithm by HWs of varying backgrounds. Smartphones and tablets have the potential to facilitate the scale-up of standard recommendations in low resource settings.

## Methods

### Structured literature reviews

In order to identify the relevant diseases to be addressed in the algorithm, estimated data on the causes of global childhood mortality and morbidity from the Child Health Epidemiology Reference Group (CHERG)[13] publications, and from the Global Burden of Disease website[14] were reviewed to assess the burden of diseases in African children. A structured literature review (SLR) was also conducted to understand the clinical presentation (accurate clinical predictors) and diseases’ distribution in children under 5 years of age (U5) attending PHCFs in developing countries, as well as appropriate POCTs for the diagnosis of the targeted diseases.

Medline (PubMed), Embase (Ovid), and the Cochrane Database of Systematic Reviews (CDSR) were explored from inception to December 31^st^ 2010, looking for articles assessing i) the prevalence of diseases and clinical features in U5 attending outpatient facilities in developing countries, and ii) the accuracy of diagnostic procedures for each of the targeted diseases. The detailed search strategy is described in Table 1. Papers involving U5 managed for acute medical conditions in ambulatory settings were selected. Studies involving only infants below 3 months of age or only adults were excluded. For prevalence of syndromes and diseases at PHCF, studies describing the clinical presentation and/or diagnoses presented by U5 attending outpatients facilities in developing countries were selected. For diagnostic procedures of targeted diseases, studies assessing accuracy of either clinical predictors or POCT were chosen. Systematic reviews addressing the questions of interest were also considered. An additional hand searching of reference lists of selected papers completed these searches. In order to better explore the accuracy of the clinical diagnosis for pneumonia, a systematic review of the literature and meta-analyses of studies assessing the diagnostic accuracy of clinical predictors was conducted, reported elsewhere (Rambaud Althaus et al, submitted).

**Table 1:** Structured literature reviews: search strategy.

	Pubmed	Embase
1	"primary health care" OR "outpatients" OR "family practice" OR "emergency service" OR "ambulatory care"	
2	"fever/etiology"[MeSH Terms] OR "fever/diagnosis"[MeSH Terms] OR "fever/epidemiology"[MeSH Terms]	
3	"developing countries"	
4	prevalence OR epidemiology OR incidence	
5	"predictive value of tests"[MeSH Terms] OR "sensitivity and specificity"[MeSH Terms] OR "reproducibility of results"[MeSH Terms] OR diagnostic test OR diagnostic tests OR "physical examination"[MeSH Terms] OR"medical history taking"[MeSH Terms]	'diagnostic accuracy'/exp OR 'predictor variable'/exp
6	"pneumonia"[MeSH Terms]	'pneumonia'/exp OR 'lower respiratory tract infection'/exp OR 'respiratory tract infection'/exp
7	“typhoid fever” [MeSH Terms]	'typhoid fever'/exp
8	“urinary tract infections”[MeSH Terms]	'urinary tract infection'/exp
9	“otitis media”[MeSH Terms]	'otitis media'/exp
10	“shigella”[MeSH Terms] OR “dysentery”[MeSH Terms]	'shigellosis'/exp
11	Filters: Infant: 1–23 months; Preschool Child: 2–5 years	'child'/exp
Prevalence	[ 1 AND ( 2 OR {3 AND 4} ) ] AND 11	
Diagnostics	6 AND 5 AND 11	6 AND 5 AND 11
	7 AND 5 AND 11	7 AND 5 AND 11
	8 AND 5 AND 11	8 AND 5 AND 11
	9 AND 5 AND 11	9 AND 5 AND 11
	10 AND 5 AND 11	10 AND 5 AND 11

### Findings of the study on causes of fever in outpatient Tanzanian children

In a recently published study on etiologies of fever conducted in outpatient clinics in Tanzania (Tanzanian fever study), clinical assessments and laboratory tests were performed in 1005 febrile children aged 2 months to 10 years (95% were U5) to establish the most probable causes of fever[15]. The distribution of diagnoses, overall and stratified by age, was taken into account to select the targeted disease included in the final algorithm. The clinical predictors for the targeted diseases identified in the Tanzanian fever study were also used to build the new algorithm (De Santis et al, in preparation).

### Algorithm construction

With the IMCI algorithm for children 2–59 months of age as departure point, the evidence retrieved from the SLRs and from the Tanzanian fever study was used to propose modifications and new recommendations when relevant, and to design a new decision tree. Diseases were included in the algorithm if they were treatable, and responsible for i) high child mortality and morbidity, ii) high attendance rate at outpatient facilities, and, iii) high antimicrobial prescription rate. Clinical features that could easily be assessed by HWs of varying background and POCT easy to deploy in low resource ambulatory settings were integrated in the classification procedures, when its use improved the classification accuracy. The treatment options proposed in ALMANACH were in line with the 2008 WHO IMCI [16] and the Tanzanian National Standard Treatment guidelines [17] since the algorithm was intended to be used in Tanzania and had to comply with local policies. Once the new algorithm was finalized, both a paper booklet and an electronic software running on android smartphones and tablets were developed. For the paper booklet, the IMCI structure in 3 steps was kept—“Assess, Classify, and Treat”—, as well as the color-coded triage system: red for conditions that require urgent referral, orange for conditions requiring specific treatment, and green for condition needing simple counseling and symptomatic home management[12].

## Results

**Fig 1:**
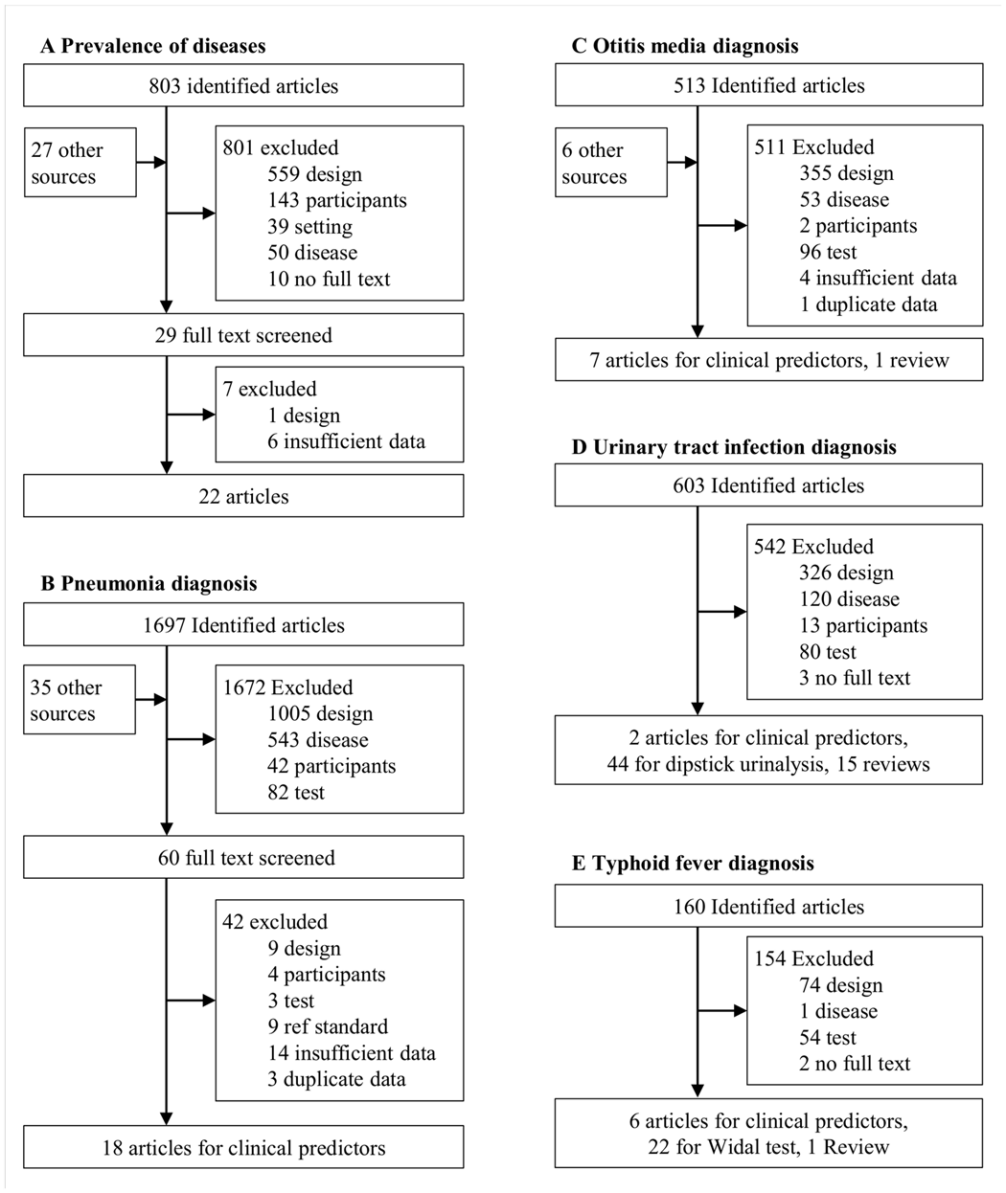
Flow diagrams of study selection process in the structured literature reviews.

Flow diagrams of studies selection for the SLRs are available in Fig 1.

All modifications made to the IMCI content based on new findings are presented in Table 2. The major changes concerned: malaria and pneumonia diagnosis; otitis media treatment; the addition of urinary tract infection (UTI) and possible typhoid fever; and a new classification entitled “likely viral infection”. The most important modifications are discussed below.

**Table 2:** Major changes in ALMANACH as compared to IMCI algorithm based on evidence and experts’ opinion.

Location	Topic	IMCI	ALMANACH	Rationale
**Management of very severe diseases section**	**Very severe diseases**	“A child with any general danger signs needs URGENT attention; **complete the assessment** and pre-referral treatment immediately so that referral is not delayed”	If the child has any general danger sign, HWs are not asked to complete the assessment of all symptoms, but rather to “**Give pre-referral treatment and REFER URGENTLY**”	To complete the assessment would delay pre-referral treatment, and impair prognosis. In presence of general danger sign, the priority is to give rapidly presumptive AB/AM treatment[42,43] and to refer to hospital, where further etiological investigations will allow adapting the treatment.
	**List of general danger signs**	“Lethargic; Convulsing; Unable to drink/breastfeed; Vomits everything; History of convulsion”	“Convulsing; Lethargic; Unable to drink/breastfeed; Vomits everything; History of convulsion; Jaundice; Cyanosis; Stiff neck; Severe pallor; Severe wasting”	Stiff neck, severe pallor, and severe wasting (assessed later on in IMCI) are part of the ALMANACH initial assessment, in order to facilitate and fasten the detection and management of very severe diseases. Jaundice and cyanosis that are strong predictors for serious bacterial diseases and severe respiratory conditions [15], have been added to the general danger signs.
	**Pre-referral treatment**	Available in the “TREAT THE CHILD” section in the middle of the booklet	Available in the “Management of very severe diseases” section in the first pages of the booklet	To facilitate and fasten the management of severe patients, the first section “Management of very severe diseases” has all assessment, classification and treatment charts together.
**Management of children with no general danger signs**	**Fever** ^§^	Fever^§^ is one of the 4 “Main symptoms”.	Fever is a crossing point in ALMANACH: different recommendations are made for children (non-severe) with or without fever^§^.	In children having no underlying chronic condition, and no danger signs, only few bacterial infections should be considered. Apart from dysentery and soft tissue infection, antibiotics are not recommended in the treatment of non-severe non-febrile conditions in ALMANACH.
	**Fever**	Classifications considered in the Fever chart are: “Very severe disease”, “Malaria” and “Measles”. *Additional classification in low malaria risk contexts*: *“Fever*, *malaria unlikely”*	Classifications considered in the Fever algorithm: “Malaria”, Acute respiratory infections, including “Pneumonia”; Diarrhea related classifications; Ear related classification; Measles; Skin infections; “UTI”, “Typhoid fever”, “Likely viral infection”	Designing a specific chart for patients with fever allows considering more fever related classifications than in IMCI, thus to address relevant non-malaria fever. This design allow also to consider “Likely Viral infections” after having excluded potentially life threatening conditions
**Febrile chart**	**Malaria**	Presumptive diagnosis of malaria for all children with fever in high malaria risk contexts	Test-based malaria diagnosis is recommended, using mRDTs in all children with fever. Antimalarials only recommended in test positive patients	The accuracy, the performance and the safety[7] of a diagnostic strategy based on mRDTs have been evaluated and demonstrated in U5.
**FebrileCough chart**	**Pneumonia**	Pneumonia diagnosis rely on increased respiratory rate (RR) above age specific threshold: 50 breath/min if aged 2–11 months; 40/min if aged 12–59 months	Pneumonia is considered in children aged 2–59 months, if they report the presence of fever and have a RR above 50 breaths/min	The need of antibiotics in children aged 2–59 months with non-severe pneumonia as defined in IMCI is questioned[76]. In children aged 12–59 months the gain in sensitivity doesn’t balance the loss of specificity for the diagnosis of pneumonia when using the threshold 40 instead of 50 breath/min. (see results section)
**Ear problem chart**	**Acute ear infection**	Oral antibiotics are recommended for “Acute ear infection” defined as either “ear pain” or “ear pus/ discharge for less than 14 days”	Oral antibiotics are only recommended for children with fever and “ear pus/ discharge for less than 14 days”	The need for antibiotics for otitis media is questioned[56]. Ear pain is a weak predictor of otitis media[53,55,77] especially in children below 2 years of age. AB are most useful for children with otitis media and ear discharge[56].
**Skin problem chart**	**Skin and soft tissue infections**	Some guidance provided in an annex and not integrated with the complaints of the main algorithm	Referral to hospital is recommended for febrile skin lesions with a size >4 cm or associated with red streaks or tender nodes, and for multiple abscesses. Local treatment and home management is recommended for impetigo and minor abscesses	Severe soft tissue infections require in hospital treatment and injectable antibiotics. Limited skin infections can be safely managed by topical treatment.
**Febrile chart for “Fever with no identified cause” after symptom charts assessment**	**Urinary tract infection**	Not considered in IMCI	Considered in non-severe febrile children, under 2 years of age, with no primary focus identified; and in children, above 2 years of age, with dysuria. Urinalysis using a dipstick is recommended for the diagnostic.	UTI is most frequent in children under 2 years of age. Above 2 years of age, the specificity of dysuria symptoms is low. The accuracy and performance of dipstick for UTI diagnosis have been demonstrated. Dipsticks for pregnancy follow-up were already broadly available in PHCFs in Tanzania; dipsticks for urinalyses were available in Health Centers.
	**Typhoid fever**	Not considered in IMCI	In non-severe febrile children above 2 years of age, with no primary focus identified, abdominal palpation is recommended. In presence of tenderness, a presumptive treatment for typhoid fever and invasive intestinal bacterial infections is recommended.	Typhoid fever and other invasive enteric infections are life threatening conditions. In low resource care facilities, HWs fear to miss these diagnoses and tend to overprescribe antibiotics to children with no identified causes of fever. In the Tanzanian fever study, abdominal tenderness was associated with invasive bacterial infections and typhoid[15], in children above 2 years of age.

AB: antibiotics, AM: antimalarials, HW: health worker, IMCI: Integrated Management of Childhood Illness, PHCF: primary health care facility, U5: children under 5 years of age, UTI: urinary tract infection.

^§^Fever is defined by either history of fever or axillary temperature above 37.5°C or child feels hot.

### Selection of syndromes or diseases to be addressed by the algorithm

Estimations of burden of diseases by CHERG[1] and IHME[14,18] reported that low respiratory tract infections/pneumonia, malaria, and diarrhea were the leading causes of child mortality in 2010, globally and in Sub Saharan Africa (SSA). These 3 infectious diseases were estimated to be responsible for more than 40% of U5 deaths in SSA. They were also the leading causes of morbidity, responsible for 41% of the total 2010 DALYs in SSA[14]. Other frequent causes of child mortality were HIV/AIDS (3.5 to 4% of U5 deaths in SSA[1,14]), meningitis (3 to 4%[1,14]), measles (1%[1,14]), and tuberculosis (0.8%[14]). In infants aged 1 to 11 months, pertussis (2.8% of deaths in 1–11months infant in SSA) and syphilis (2.3%) were also frequent causes of death[14]. In children aged 1 to 4 years, typhoid fever was estimated to be responsible for 0.6% of both DALYs and deaths, and bacterial skin diseases for 0.7% of DALYs, and 0.2% of deaths[14].

The SLR identified 22 articles assessing either symptoms or diagnoses distributions, or both, in children attending outpatient facilities in developing countries. In all selected papers assessing symptoms, fever (by history or measured, hereafter referred as fever), cough and diarrhea were the most frequent symptoms reported, respectively by 65 to 93%[19–24], 44 to 82%[19–21,23,24], and 22% to 45%[19–21,23] of children. Diseases of potential bacterial origin reported in the studies retrieved by the SLR were: pneumonia (reported in 5 to 30% of children[15,19–21,24–31]), typhoid fever (3 to 13%[15,24,26]), dysentery (3 to 12% [19,28,29]), otitis media (2 to 12%[7,19–21,28–31]), UTI (1 to 7%[15,24,28,30,32,33]); and meningitis (0 to 3%[19,25,28]). Tonsillitis was reported in 1% of 1005 children in the Tanzanian fever study; all had a negative streptococcal diagnostic test[15]. Another study reported tonsillitis or pharyngitis in 10% of the children, but no streptococcal test was performed[30].

Among the bacterial infections frequently reported, only typhoid fever, UTI and tonsillitis were not yet addressed in IMCI. The fear of these 3 infections is often a reason to prescribe antibiotics in low resource setting. With regards to tonsillitis, early recognition and treatment of streptococcal tonsillitis is of high importance to prevent rheumatic fever and its complications, but prevalence of group A β-hemolytic streptococcus is much lower in U5 than in older children[34], and close to zero in children under 2 years of age[35]. Moreover, acute rheumatic fever and rheumatic heart disease are rare in U5[36,37]. Therefore addressing streptococcal tonsillitis in the management of U5 was considered not to be necessary. UTI and typhoid fever were thus selected to be addressed in the new algorithm, together with the other diseases already addressed in IMCI.

### Identification of severe illnesses

In the IMCI algorithm, urgent referral to hospital is recommended in the presence of any of 5 general danger signs (difficulty in drinking, repeated vomiting, had convulsion, lethargy or unconsciousness, convulsing) or in presence of any of the 8 symptom-related danger signs (danger sign related to fever: stiff neck; related to cough: stridor or chest indrawing; related to measles features: clouding of cornea or extensive mouth ulcers; related to malnutrition: severe wasting or oedema of both feet; related to pallor: severe palmar pallor). The usefulness of these IMCI referral criteria were evaluated in two studies[38,39]. These studies estimated the accuracy of the presence of any of the danger signs to predict hospital referral. Estimates of sensitivity and specificity were 46% and 79% respectively in the Kenyan study [38] and 86% and 64% respectively in the study conducted in Bangladesh [39]. In the Kenyan study, accuracy of these criteria to predict death in admitted U5 patients was also assessed (sensitivity 89%, specificity 44%)[38]. A systematic review for children in developed countries[40] has also identified reduced consciousness, convulsions, cyanosis, rapid breathing, and slow capillary refill as the strongest predictors of severe illness. Meningeal irritation was also a strong predictor of serious bacterial infection in 3 reported studies [likelihood ratio (LR) ranging from 2.57 to 275] [40]. While reviewing the underlying evidence used to build the recommendations for referral in IMCI, only very few studies were identified. As for IMCI, one of the main aims of the present algorithm is to allow early identification and referral of children with severe conditions and serious bacterial infections; therefore, to ensure good sensitivity, all the IMCI referral criteria were kept although underlying evidence was scarce, and 2 signs predicting serious infections were added to the IMCI general danger signs, i.e. cyanosis that is broadly recognized as a sign of severe hypoxemia [40] and jaundice that was shown to be predictive of bacterial disease in the Tanzanian fever study[15] and can also be associated with severe malaria [41]. In order to improve and fasten the identification of severe patients, all general danger signs, were grouped together with stiff neck, severe wasting, and severe pallor at the beginning of the assessment chart, instead of having some of them included in the branches for each syndrome. Danger signs related to specific symptoms were kept within the symptoms related charts, such as chest indrawing with cough, or tender swelling behind the ear with ear problem. Children with general danger signs are classified as having ‘Very severe disease’. These children with general danger signs are at risk of severe sepsis. In ALMANACH, acknowledging that early antimicrobials can improve the outcome of these children prognosis[42,43], and realizing that the full assessment would not modify this recommendation, a separate management chart was developed for patients with danger signs, allowing prompt presumptive treatments, and skipping assessment tasks that would only delay the rapid management and referral these children require.

### Malaria diagnosis

Decline in the proportion of fevers due to malaria [44] together with the availability of easy-to-use, reliable POCTs—i.e. mRDTs—have driven the WHO recommendations to shift in 2010 from presumptive to test-based malaria case management[45]. The safety of a mRDT-based malaria case management in U5 has been demonstrated[7–10,46–48]. Several African countries have now changed their malaria diagnosis policy and adopted the use of mRDTs in their national programs. Following the new WHO malaria treatment guidelines, the use of mRDTs was integrated in present algorithm. mRDTs were also recently added officially to the WHO/UNICEF generic IMCI algorithm[49].

### Pneumonia diagnosis

In a recent meta-analysis of clinical predictors for radiological pneumonia (Rambaud Althaus et al, submitted), the clinical features with the higher pooled LR were respiratory rate ≥50 breaths/min (LR 1.90; 95%CI 1.45–2.48), grunting (1.78; 1.10–2.88), lower chest indrawing (1.76; 0.86–3.58), and nasal flaring (1.75; 1.20–2.56). The best features to rule out the diagnosis (having the lowest pooled LR) were: no history of fever (0.53; 0.41–0.69), and respiratory rate ≥40 breaths/min (0.43; 0.23–0.83). Cough had also a low but heterogeneous LR (0.30; 0.09–0.96). The IMCI criterion for pneumonia classification, i.e. age-related fast breathing (≥50/min from 2 to 11 months, and ≥40/min from 12 to 59 months) showed low diagnostic performance in the meta-analysis, both to rule in the disease (presence of fast breathing had a pooled LR of 1.55 (0.44–5.42)) and to rule it out (absence of fast breathing had a pooled LR of 0.63 (0.16–2.55)). In the Tanzanian fever study, the best predictors to rule in radiological pneumonia among all febrile children were difficult breathing (LR 7.9, 2.8–22.1), chest indrawing (7.1; 2.9–17.6), nasal flaring (7.0; 2.5–19.4), respiratory rate ≥50/min (6.1; 3.5–10.4) and abnormal chest auscultation (5.5; 3.7–8.1). No feature was good at ruling out the diagnosis. In the present algorithm, in the absence of a reliable point-of-care diagnostic test, we decided to combine the best available clinical predictors (history of fever, cough, difficult breathing and fast breathing), except nasal flaring and grunting, and abnormal chest auscultation because most IMCI trained clinicians are not familiar with these features. Chest indrawing was kept but to decide on referral to hospital rather than to diagnose pneumonia, because of the relatively high proportion of these children that harbor hypoxemia[50] Regarding fast breathing, because using an age-related threshold did not improve the diagnostic test accuracy in the meta-analysis (Rambaud Althaus et al, submitted), a single threshold of ≥50/min for all age groups was chosen; 50/min rather than 40/min was chosen to ensure a reasonable specificity, knowing that most of pneumonias in young children are due to viruses[51]. The recommendation in the present algorithm is thus to prescribe antibiotics for pneumonia to children with [history of fever or elevated temperature] AND [cough or difficult breathing] AND respiratory rate ≥50/min, regardless of the malaria test result.

### Otitis Media

In the SLR 7 articles and a systematic review that addressed the question of the accuracy of symptoms and signs for the diagnosis of otitis media were retrieved[52]. In these studies, some otoscopic signs were strongly associated with otitis media diagnosis[52], but in low resource settings otoscopy is not available in ambulatory care. Other symptoms, such as earache, ear rubbing, and fever, although reported as associated with otitis media in 4 old studies (LR 3.03 to 7.3[53–55]), were not associated with this diagnosis when reported by parents of children aged 6 to 36 months attending primary care offices in a more recent study(52). Otitis media is often a self-limiting condition in young children. The 2010 Coker[52] and Sanders’ Cochrane[56] reviews, looking at available evidence of the benefit of antibiotic treatment for otitis media, report that there is little benefit (compared to placebo) and no evidence that antibiotics reduce complications or recurrence[52,56]. An individual patient data meta-analysis from 6 randomised trial reported that antibiotics were more beneficial in children aged less than 2 years with bilateral otitis media, and in those with both otitis media and otorrhoea. In children with otorrhoea, 60% of controls and 25% of those on antibiotics still had pain, fever or both at 3–7 days, with a rate difference of -36% (95%CI -53% to -19%) and a number needed to treat of 3, whereas in children without otorrhoea the rate difference and NNT were respectively -14% (-23% to -5%) and 8[57].

Otitis media being often a self-limiting condition in young children, in the absence of accurate non-otoscopic clinical predictors the new algorithm propose to limit antibiotic prescription to children presenting with ear discharge.

### Urinary tract infection

Two articles and 12 reviews assessing the accuracy of clinical predictors for the diagnosis of UTI in children were retrieved from the SLR. No additional article since the most recent review published in 2007 was found[58]. The following predictors were identified: temperature >40°C (2 studies, LR 3.3; 1.3–8.3[59] and LR 3.2; 0.7–15.6[60]), jaundice (LR 2.1; 0.3–17.4)[61], and suprapubic tenderness (LR 4.4; 1.6–12.4)[62]. The absence of another source of fever on examination increased the probability of UTI (3 studies, summary LR 2.8; 1.9–4.3)[58]. Among children ≥2 years, abdominal pain (LR: 6.3; 2.5–16.0) [61], dysuria (LR 2.4; 1.8–3.1)[63] and new-onset of urinary incontinence (LR 4.6; 2.8–7.6)[63] also increased the probability of UTI.

In the Tanzanian fever study, the following predictors to rule in UTI were found: pollakiuria (LR 3.5; 1.4–8.8), temperature >40°C (3.1; 1.4–7.1), fever for more than 3 days (2.1; 1.2–3.6) and age<2 years (1.4, 1.22–1.57); the best predictors to exclude UTI were: age≥3 years (LR 0.22; 0.07–0.66), headache (0.27; 0.04–1.89) and diarrhea (0.33; 0.08–1.32) (De Santis et al, in preparation). Based on these predictors, several national and international guidelines recommend to consider this condition in febrile children below 2 years of age, with no obvious cause of fever[32,64]. No symptom or sign, nor combination of them is predictive enough in this age group to appropriately identify children with UTI. The gold standard (urine culture) is generally not available in low resources ambulatory setting. Urinalysis with urine dipsticks detecting leucocyte esterase and nitrite has been evaluated in many settings: 4 systematic reviews with meta-analyses estimated sensitivities for leucocyte esterase and/or nitrites to be 81%[65], 88%[66,67], and 93%[32] and specificities 72%[32], 79%[67], 93%[66] and 97%[65]. A dipstick urinalysis negative for both nitrites and leukocyte esterase had a LR of 0.2 (95% CI, 0.16–0.26)[58]. With either leucocyte esterase or nitrite positive the LR was 6.1 (95% CI, 4.3–8.6), increasing to 28 (95% CI, 17–46) when both leucocyte esterase and nitrite were positive[65]. In 2005, the WHO department of Child and Adolescent Health and Development recommended the use of urinalysis by urine dipstick for the diagnosis of UTI in children wherever dipstick were feasible[64]. With the implementation of the WHO focused antenatal care guidelines, urine dipstick for proteinuria detection have been implemented and are thus available in PHCFs in many African countries. Based on the good diagnostic performance of urine dipstick, and it’s feasibility in low resource setting, the new algorithm proposes to perform urine dipstick for the diagnostic of UTI in the patients at higher risk of UTI, i.e. children below 2 years of age having fever with no cause identified during the assessment (but regardless of the malaria test result, due to the possibility that the parasites might only correspond to an incidental infection and not the actual cause of the acute illness). For children from 2 to 5 years of age, only those complaining of dysuria are proposed a dipstick urinalysis. Antibiotic treatment for UTI is recommended when either leucocyte esterase or nitrite, or both are positive.

### Typhoid fever

Regarding the diagnosis of enteric fever, 6 articles assessing clinical predictors of enteric fever were retrieved [68–73]. Only 2 were conducted in outpatients: one included patients above 15 years of age[71] and the other patients above 4 years of age[70]. None of the studies thus included our target population of U5 outpatients. In the Tanzanian fever study[15], the following predictors to rule in typhoid where identified: liver pain (LR 9.8; 2.7–35.5), abdominal tenderness (7.0; 3.3–15.2), jaundice (6.2; 3.1–12.4) and age >2 years (2.0; 1.6–2.4). To rule out typhoid, only ‘not during rainy season’ was predictive (LR 0.50; 0.27–0.92) (De Santis et al, in preparation). Jaundice being already included as danger sign and liver pain being difficult to assess in a child, the new algorithm recommends looking for abdominal tenderness in children ≥2 years of age having fever with no cause identified during the child’s assessment (regardless of the malaria test result). When present, antibiotic treatment for typhoid fever is indicated.

### Likely viral infection

Likely viral infection is a classification proposed in the present algorithm that does not exist in IMCI. Unnecessary antibiotics are often prescribed in febrile children by HWs when they do not manage to reach a diagnosis after their assessment, because they fear to have potentially missed a life-threatening bacterial infection. Because in the present algorithm most of the frequent bacterial infections have been assessed for, the probability that the child is still suffering from one is low if all findings are negative. Therefore, in the absence of danger signs, cough or fast breathing, diarrhea, ear discharge, symptoms of measles, infected skin lesion, abdominal tenderness, a positive dipstick urinalysis and a positive malaria RDT, the child is classified as having a “Likely viral infection”. HWs are then proposed to withhold antibiotics and antimalarials, prescribe symptomatic treatment for fever if any, and advise the caretaker on when to come back if symptoms persist or worsen.

### Design of the algorithm

**Fig 2:**
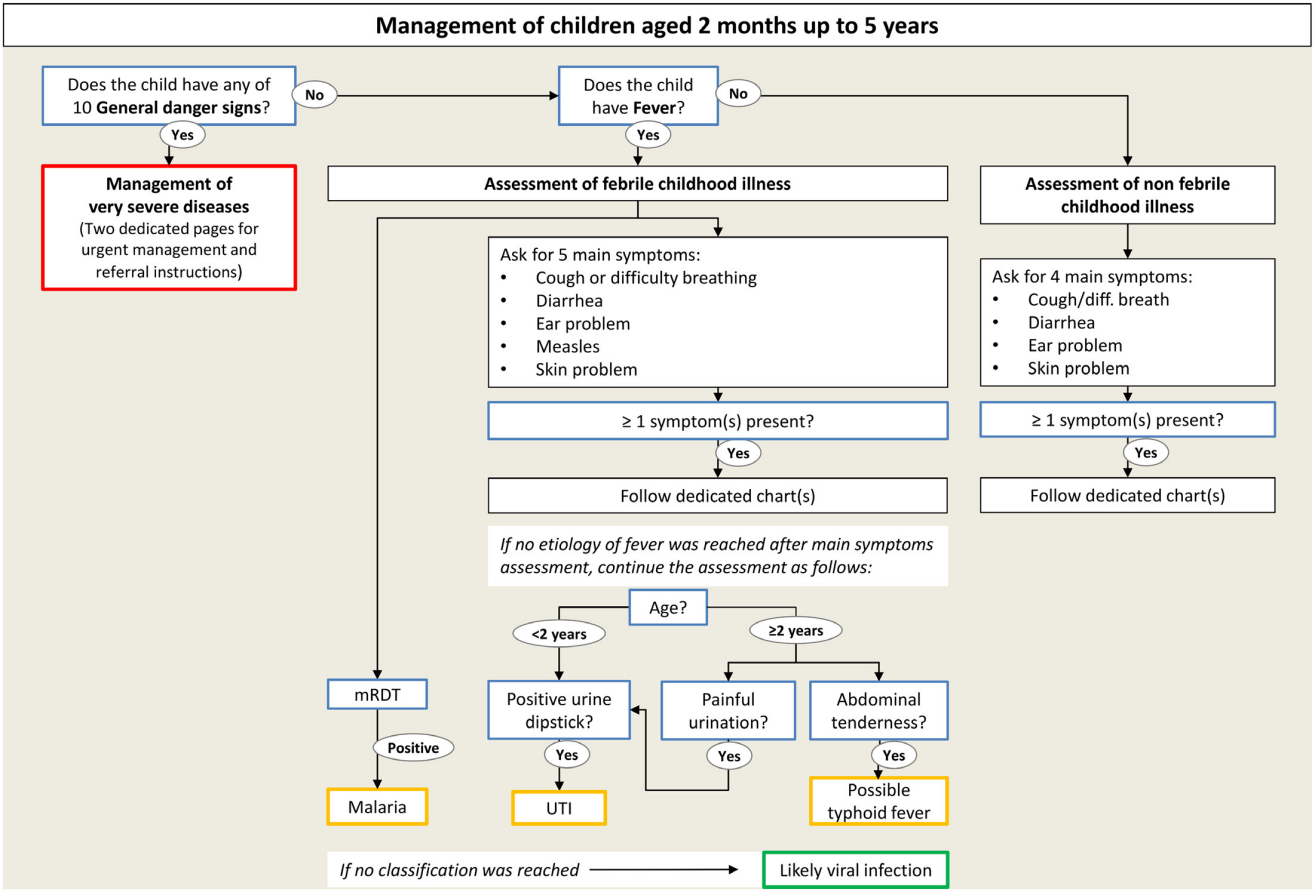
Overview of ALMANACH's structure. mRDT: malaria rapid diagnostic test. UTI: urinary tract infection.

Based on the modifications and adjunctions to IMCI that were retained, a new algorithm for the management of childhood illnesses (ALMANACH) was designed. Efforts were made to keep the ALMANACH structure simple and graphically easy to follow by HWs. Therefore the IMCI 3 steps assessment and color codes were kept. However, in order to increase the number of conditions addressed, ALMANACH has been divided into 3 charts. The first chart provides recommendations for assessment of general danger signs and management of severe patients, the second chart provides recommendations for patients with fever, and the last one for patients without fever (see Fig 2 for an overview of ALMANACH’s structure). This 3-charts structure allows i) fastening the assessment and management of severe children, for whom all recommendations are available in the very first part of the algorithm and ii) a more comprehensive and specific assessment of children, with pneumonia, malaria, UTI and typhoid fever being considered only in febrile children. In the IMCI algorithm, fever is one of the main symptoms. The IMCI fever box only considers malaria and measles for a child with acute fever (≤7 days) without danger signs. Within ALMANACH, the aim was to address non-malaria fever causes. Although parts of the algorithms, such as the diarrhea chart, are the same in both the ‘febrile’ and ‘non-febrile’ algorithms, replacing the fever box by a full algorithm for children with fever or history of fever was necessary to propose meaningful considerations of fever causes in sequential pathways, allowing considering some conditions in a subset of patients only. In addition, this categorization allowed limiting antibiotic treatment in children without fever or history of fever.

**Fig 3:**
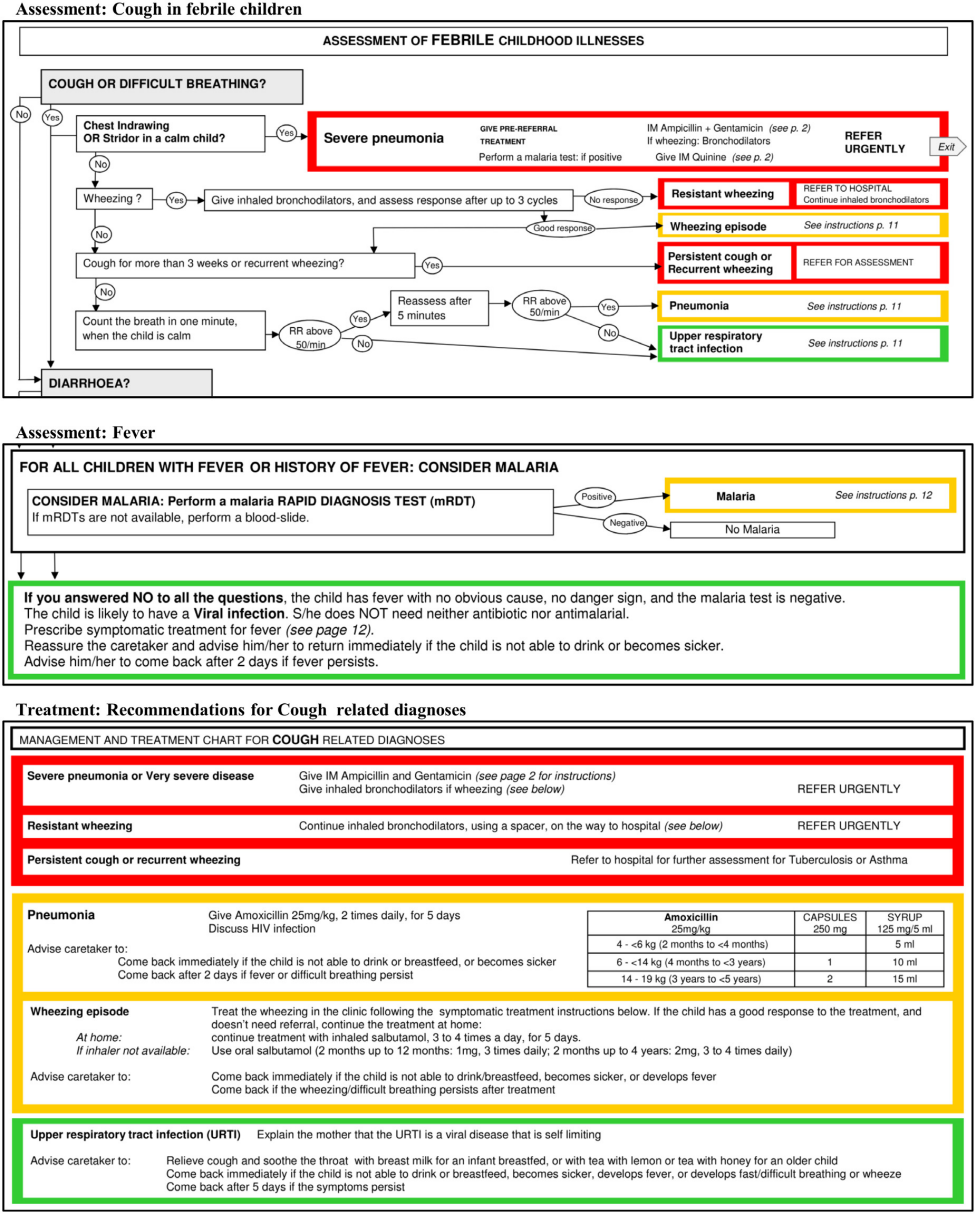
Samples of ALMANACH in paper format.

**Fig 4:**
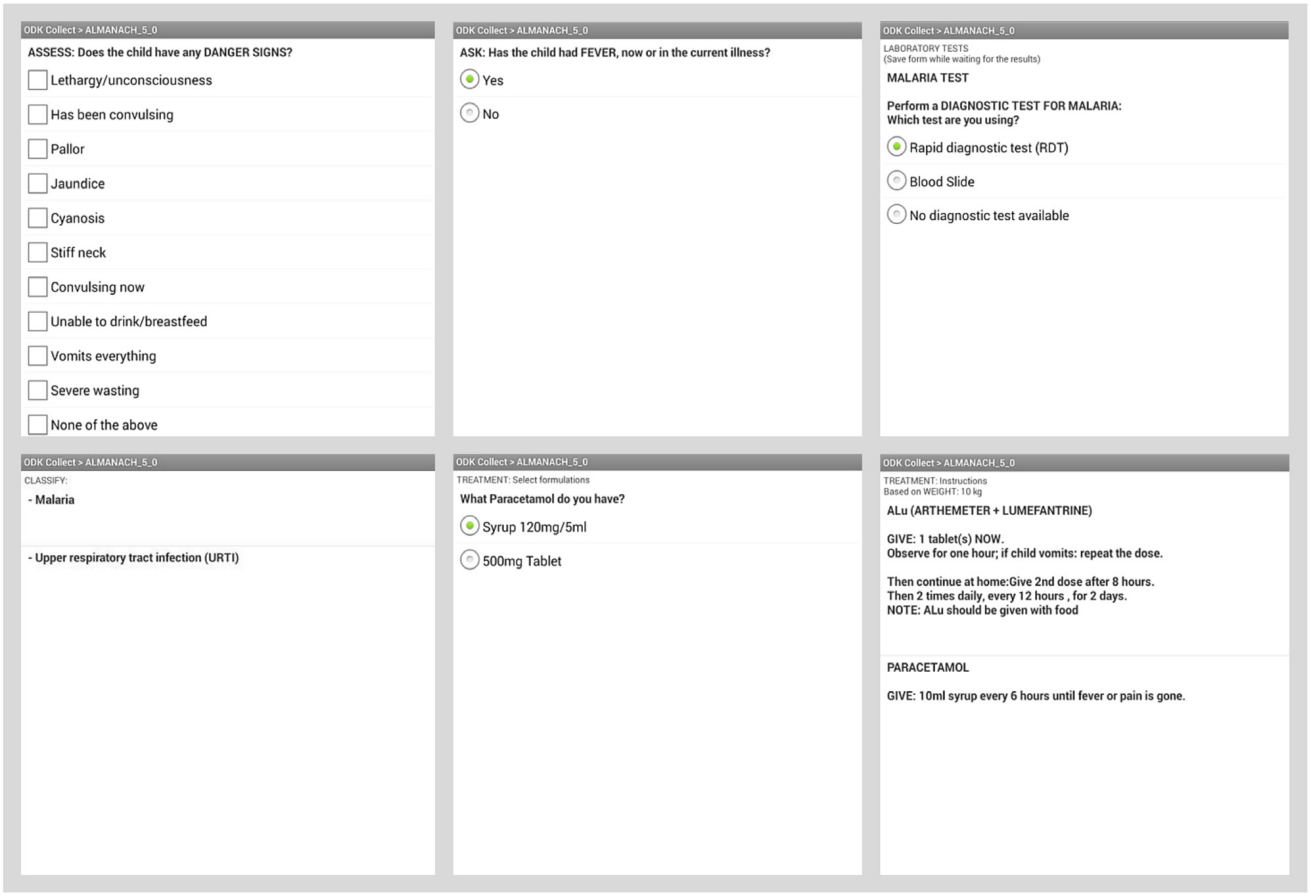
Samples of ALMANACH in electronic format.

ALMANACH was first designed as a paper booklet (Fig 3 and S1 Fig), it was then developed as an android application for smartphones, coding the different steps of the algorithm into a Java-Rosa X form run by OpenDataKit and OpenMRS software[74,75]. The electronic ALMANACH (e-ALMANACH) guides HWs through the child’s assessment up to the classification and treatment recommendations (Fig 4). Treatment dosages are computed according to the body weight or age when weight is not available. Moreover e-ALMANACH collects in real time information on child demographic characteristics, disease classification and treatment prescribed. This information is stored by the mobile device, can be sent to a server and feed health information systems.

## Discussion

The aim of ALMANACH is to provide guidance to health workers on antimicrobial prescription, in order to treat only children aged 2 to 59 months who will potentially benefit from them. Apart from malaria, IMCI was not directly addressing causes of fever, leaving HWs with their fear of life-threatening conditions once malaria was ruled out by mRDT. On the other hand, viral infections that represent the vast majority of the causes of fever in U5 children[15] are never explicitly mentioned or proposed as diagnosis in IMCI, giving a wrong impression to health workers that bacterial infections are frequent and that children should often be prescribed antibiotics. Using the best available and feasible diagnostic procedures for the main causes of acute illness in children attending PHCFs, the present new algorithm should address most of the concerns of HWs regarding bacterial infections and remind them that children often suffer from self-limited viral conditions that do not warrant any specific treatment beside antipyretics. By providing tools to rule out malaria, UTI, and typhoid fever and by proposing a new ‘Likely viral infection’ classification, the use of ALMANACH has thus the potential to improve the health outcome of febrile children and at the same time decrease unnecessary antimalarial and antibiotic prescriptions. The content of ALMANACH was based on literature reviews and expert opinions. The level of evidence provided by the literature was generally low and no formal process was followed to reach a broad expert consensus. Within the current project, only the POCTs currently available in low resource settings were considered, constraining the new algorithm to rely mostly on the best available simple clinical predictors. To further improve the quality of the management of pediatric illnesses and the rational use of medicines, accurate and affordable POCTs for bacterial or even viral infections are highly needed.

While broadening the spectrum of diseases to be addressed, the algorithm became more complex than IMCI. This might be an issue for the targeted audience, i.e. HWs of different background working in low resource ambulatory settings. In order to facilitate understanding and usability of the decision chart, the 3 steps IMCI structure (Assess, Classify and Treat) and the color coded triage, already known by IMCI trained HWs, were kept. Electronic algorithms, by guiding HWs step by step through the algorithm, allow to using a more complex structure with lower risk of misuse. The electronic version of ALMANACH running on smartphones and tablets was designed to address these needs.

Although ALMANACH broadened the spectrum of diseases addressed in the algorithm, some aspects of childhood illness were left uncovered. Indeed ALMANACH does not provide recommendations for the management of chronic or non-infectious conditions. Within the current project, only identifiable and treatable acute infections were targeted because the objective was to improve the use of antimicrobials in order to tackle both the risk of resistance development due to their overuse and the high childhood mortality related to infectious diseases. Because the algorithm was meant for remote PHCF, full algorithms for the management of severe conditions, for HIV-infected and/or malnourished children were not developed; only recommendations on how to identify children suffering from these conditions and advice to refer them to the second level of care were provided. We foresee that developing and integrating additional algorithms for the management of these conditions, but also for other patients, either at primary or secondary level of health care systems, would allow further improvement of the quality of health services, but also better acceptability of the tool by HWs. However this was out of the scope of the present project. The paper and electronic ALMANACH have the potential to improve the management of the sick child. This has been demonstrated in a recently completed feasibility study, which showed the ALMANACH algorithm to improve health outcome of children managed with this tool and to drastically reduce antibiotic prescription (Shao et al, companion paper submitted). These results were obtained in two settings, urban and rural, albeit with a limited number of patients enrolled. They do not represent a definite validation of ALMANACH, but show great promise and should invite researchers to further explore the potentials of this new approach for a rational management of children aged 2–59 months. Further improvement could be brought by integrating other POCT detecting key pathogens once they become available, or even better, by integrating host biomarkers able to predict children in need of antibiotics or at risk of dying.

## Supporting Information

S1 Fig ALMANACH: A new ALgorithm for the MANAgement of CHildhood illnesses for children aged 2 months up to 5 years. (PDF)
