# Supplementary material for: Managing the Sick Child in the Era of Declining Malaria Transmission: Development of ALMANACH, an Electronic Algorithm for Appropriate Use of Antimicrobials
Source: PLoS One. 2015 Jul 10;10(7):e0127674. doi: 10.1371/journal.pone.0127674 (PMC4498609; doi:10.1371/journal.pone.0127674)

ALMANACH:

A new ALgorithm for the MANAgement of CHildhood illnesses

For children aged 2 months up to 5 years

PeDiAtrick project 2009 - 2012

HOW TO USE **ALMANACH**

1. Check for GENERAL DANGER SIGNS: If any general danger sign present: give PRE-REFERRAL TREATMENT and REFER urgently to hospital — P 1-2
2. If no danger sign are present: Assess the presence of fever:
   - For a child with febrile disease: Follow instructions in 'ASSESSMENT OF FEBRILE ILLNESS' chart — P 3-5
   - For a child with no fever nor history of fever in the current illness: Follow instructions in 'ASSESSMENT OF NON FEBRILE ILLNESS' chart — P 7-8
4. TREAT THE CHILD according to diagnoses identified in the assessment chart — P 10-17

TABLE OF CONTENTS

Assessment and treatment for VERY SEVERE DISEASES
Check for GENERAL DANGER SIGNS…………………………………………………….……………..... 1
Instructions for pre-referral treatment for VERY SEVERE DISEASE ..….……………………….... 2

ASSESSMENT AND CLASSIFICATION
Does the child have fever? ……………………………………………………………………… 1
Assessment of FEBRILE childhood illnesses
Does the child have COUGH or DIFFICULT BREATHING?……………………………….. ………………..... 3
Does the child have DIARRHOEA?...…………………..... 3
Does the child have an EAR PROBLEM?...………………….... 4
Has the child had MEASLES now or in the past 3 months? ……………………………………….... 4
Does the child have INFECTED SKIN LESION? ……………………………..…………………..... 4
For children with no identified causes of fever……………………………..……………………...... 5
Assessment of NON-FEBRILE childhood illnesses:
Does the child have cough or difficult breathing? ……………………………….……………….... 7
Does the child have DIARRHOEA? ……………………………………………….……………….... 7
Does the child have an EAR PROBLEM? …………………………………………….……………...... 8
Does the child have INFECTED SKIN LESION? ……………………………..…………………..... 8

MANAGEMENT AND TREATMENT
Table of contents……...……………………………………………………..…………………...... 10
Management of COUGH related diagnoses
Severe pneumonia, Persistent cough or Recurrent wheezing, Pneumonia,
Upper respiratory tract infection, Symptomatic treatment for wheezing and cough……………………...... 11
Management of FEVER related diagnoses
Persistent fever, Malaria, Urinary tract infection, Abdominal infection
Symptomatic treatment for fever…………………………...……………………………...……12
Management of DIARRHOEA related diagnoses
Severe dehydration, Severe persistent diarrhoea, Persistent diarrhoea, Dysentery,
Acute diarrhoea with some dehydration, Acute diarrhoea with no dehydration…………………….....…. 13
Hydration plan A, B and C……………………………………………………………......….. 14
Management of EAR related diagnoses
Mastoiditis, Acute febrile ear discharge, Non febrile ear discharge,
Chronic ear discharge, Acute ear infection, Clear the ear by dry wicking……………………….....……...15
Management of ANAEMIA ……………………………………………………………………...……..15
Management of MEASLES related diagnoses
Severe measles, Measles with mouth or eye complication, Measles…………………………...……..16
Management of SKIN related diagnoses
Severe soft tissue infection, Soft tissue infection, Impetigo or minor abscess…………………...……...16

ADDITIONNAL INFORMATION
Use of a spacer…………...…………………………………………………………………...……………...17
Weight-for-age charts……………………………………………………………………...…………………17

MANAGEMENT OF **VERY SEVERE DISEASES**

CHECK FOR GENERAL DANGER SIGNS

Is the child:

convulsing now?

Yes → **Give:** Anticonvulsant (Diazepam) *See instructions p.2*

AND

No ↓

having any General Danger sign?

LOOK
Lethargic/unconscious
Severe pallor
Jaundice
Cyanosis
Stiff neck
Severe wasting

ASK
Unable to drink/breastfeed
Vomits everything
Had convulsions (in the current illness)

Yes →

Very severe disease:

The child needs **URGENT REFERRAL TO HOSPITAL**

Give PRE-REFERRAL TREATMENT:

First dose of IM Ampicillin + Gentamicin
IM Quinine
Low blood sugar prevention

See instructions p.2

AND

R E F E R U R G E N T L Y *Exit*

No ↓

Does the child have **some Pallor?**

Yes → **Anemia** *See instructions p.15*

No ↓

Does the child have **Fever ?**
By history
Or feels hot
Or axillary's temperature above 37°5C

Yes → Duration of fever?

7 days or more → **Persistent fever** → Refer for assessment *Exit*

Less than 7 days → Go to page 3: Assessment of febrile acute illnesses

No → Go to page 7: Assessment of acute non febrile illnesses

Exit *If you reach this sign, you should refer the child witout completing the assessment.*
If nothing is specified you have to complete the entire assessment before prescribing appropriate treatment.

ANTIBIOTIC: AMPICILLINE (50 mg/kg) + GENTAMICIN (7.5 mg/kg)

Ampicilline for Very severe diseases and Severe pneumonia

Preparation Check the vial: AMPICILLINE 500mg/0.4ml. Dilute the 500 mg vial in 2.1ml of sterile water. You have now 2.5ml of a solution with 200mg/ml.

Where there is a **strong suspicion of meningitis,** the **dose of ampicillin** can be **increased 4 times.**
IF REFERRAL IS NOT POSSIBLE OR DELAYED, repeat the ampicillin injection every 6 hours.

Gentamicin for Very severe diseases and Severe pneumonia

Preparation Check the vial: GENTAMICIN 40mg/ml, 2ml. You have 80mg of Gentamicin in a 2ml solution. IF REFERRAL IS NOT POSSIBLE OR DELAYED, repeat the gentamicin injection once daily.

**AMPICILLIN 500 mg vial**	
4 - <6 kg (from 2 to <4 months)	1 ml
6 - <10 kg (4 to <12 months)	2 ml
10 - <14 kg (12mths to <3 years)	3 ml
14 - 19 kg (3 years to <5 years)	5 ml

**GENTAMICIN 80mg in 2ml**	
4 - <6 kg (2 to <4 months)	0.5-1.0 ml
6 - <10 kg (4 to <12 months)	1.1-1.8 ml
10 - <14 kg (12 months to <3 years)	1.9-2.7 ml
14 - 19 kg (3 years to <5 years)	2.8-3.5 ml

QUININE (10mg/kg)

Preparation Check the ampoule: do you have: - QUININE 150mg/ml (300mg in 2 ml)
or - QUININE 300mg/ml (600mg in 2 ml)

IF REFERRAL IS NOT POSSIBLE OR DELAYED, The child should remain lying down for one hour. Repeat the quinine injection 8 hourly, until the child is able to take oral antimalarial, but not more than one week.

** quinine salt*

**INTRAMUSCULAR QUININE**	**150mg*/ml**	**300mg*/ml**
4 - <6 kg (2 to <4 months)	0.4 ml	0.2 ml
6 - <10 kg (4 to <12 months)	0.6 ml	0.3 ml
10 - <12 kg (12 mths to <2 years)	0.8 ml	0.4 ml
12 - <14 kg (2 years to <3 years)	1.0 ml	0.5 ml
14 - 19 kg (3 years to <5 years)	1.2 ml	0.6 ml

PREVENT LOW BLOOD SUGAR

If the child is able to breastfeed*:* Ask the mother to breastfeed the child.
If the child is not able to breastfeed but is able to swallow: Give expressed breast milk or a breast-milk substitute.
If neither of these is available, give sugar water*. Give 30 - 50 ml of milk or sugar water* before departure.
If the child is not able to swallow: Give 50 ml of milk or sugar water* by nasogastric tube.

** <u>To make sugar water:</u> Dissolve 4 level teaspoons of sugar (20 grams) in a 200-ml cup of clean water.*

ANTICONVULSANT: DIAZEPAM (0.5 mg/kg)

Instructions Turn the child to his/her side and clear the airway. Avoid putting things in the mouth.
Give diazepam solution per rectum using a small syringe without a needle (like a tuberculin syringe) or using a catheter.
Check for low blood sugar, then treat or prevent.
Give oxygen and REFER.
If convulsions have not stopped after 10 minutes repeat diazepam dose once.

**DIAZEPAM**	10mg/2mls
<5 kg (<6 months)	0.5 ml
5 - <10 kg (6 to <12 months)	1 ml
10 - <14 kg (12 months to <3 years)	1.5 ml
14 - 19 kg (3 years to <5 years)	2 ml

ASSESSMENT OF FEBRILE CHILDHOOD ILLNESSES

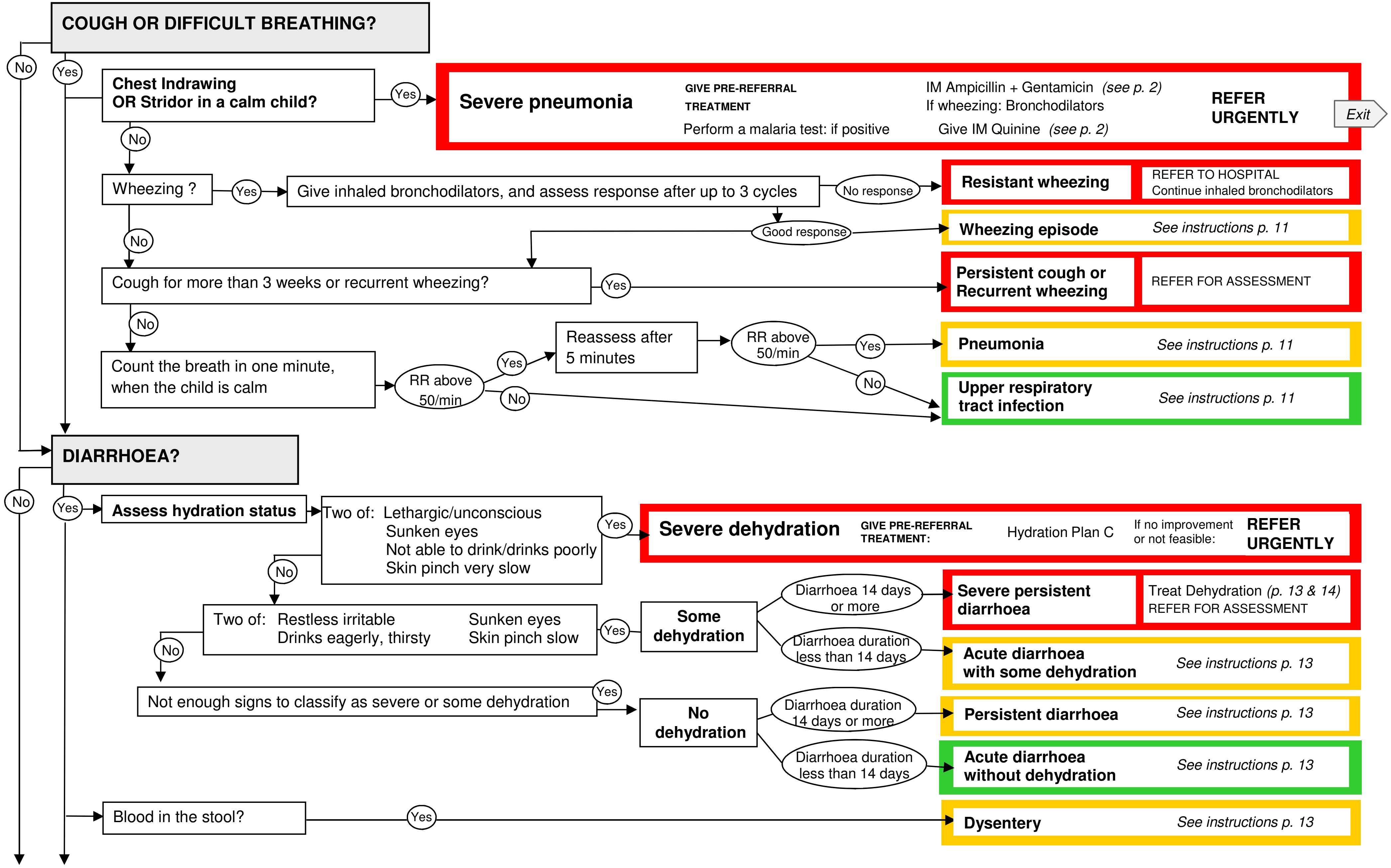

Complete assessment of febrile illnesses p. 4

EAR PROBLEM?

- No → MEASLES Now or within last 3 months?
- Yes →
  - **Tender swelling behind the ear**
    - Yes → **Mastoiditis** — GIVE PRE-REFERRAL TREATMENT: IM Ampicillin + Gentamicin *(see p. 2)*; Paracetamol for pain. Perform a malaria test: if positive: Give IM Quinine *(see p. 2)*. **REFER URGENTLY** → *Exit*
    - No ↓
  - Pus draining from the ear
    - Yes → Less than 14 days → **Acute ear discharge** — *See instructions p. 15*
    - Yes → 14 days or more → **Chronic ear discharge** — *See instructions p. 15*
    - No ↓
  - Ear pain
    - Yes → **Acute ear infection** — *See instructions p. 15*
    - No → UNCLASSIFIED EAR PROBLEM: No need for antibiotics

MEASLES Now or within last 3 months? Generalized rash

+ one of the following:
Cough, Runny nose, Red eyes

- No → INFECTED SKIN LESION OR LUMP?
- Yes →
  - **Clouding of cornea ?**
    or Deep or extensive mouth ulcers ?
    - Yes → **Severe complicated measles** — GIVE PRE-REFERRAL TREATMENT: IM Ampicillin + Gentamicin *(see p. 2)*; Vit A, Tetracycline eye o. Perform a malaria test: if positive: Give IM Quinine *(see p. 2)*. **REFER URGENTLY** → *Exit*
    - No ↓
  - Pus draining from the eye or Mouth ulcer
    - Yes → **Measles with complications** — *See instructions p. 16*
    - No → **Measles** — *See instructions p. 16*

INFECTED SKIN LESION OR LUMP?

- No ↓
- Yes →
  - **Size above 4cm? Multiple abscesses?**
    Associated with red streaks or tender nodes?
    - Yes → **Severe soft tissue or muscle infection** — GIVE PRE-REFERRAL TREATMENT: Oral Cloxacillin *(see p. 16)*. **REFER URGENTLY**
    - No ↓
  - Is the lesion red, tender, warm, with pus or crusts?
    - Yes → **Impetigo or minor abscess** — *See instructions p. 16*
    - No → UNCLASSIFIED SKIN CONDITION: No need for antibiotics

Complete assessment of febrile illnesses p. 5

ASSESSMENT OF FEBRILE CHILDHOOD ILLNESSES

After this assessment **if the child has fever with no identified cause, perform the following:**

For children aged 2 months up to less than 2 years: → Urine dipstick

- Leucocytes OR Nitrites positive → **Urinary tract infection (UTI)** — *See instructions p. 12*
- Leucocytes AND Nitrites negative → **No Urinary tract infection**

For children aged 2 years up to 5 years: → Abdominal palpation (If dysuria → Urine dipstick)

- Abdominal tenderness → **Possible intestinal bacterial disease** — *See instructions p. 12*
- Normale → **Bacterial disease unlikely**

FOR ALL CHILDREN WITH FEVER OR HISTORY OF FEVER: CONSIDER MALARIA

CONSIDER MALARIA: Perform a malaria RAPID DIAGNOSIS TEST (mRDT)
If mRDTs are not available, perform a blood-slide.

- Positive → **Malaria** — *See instructions p. 12*
- Negative → No Malaria

If you answered NO to all the questions, the child has fever with no obvious cause, no danger sign, and the malaria test is negative.
The child is likely to have a **Viral infection.** S/he does NOT need neither antibiotic nor antimalarial.
Prescribe symptomatic treatment for fever *(see page 12).*
Reassure the caretaker and advise him/her to return immediately if the child is not able to drink or becomes sicker.
Advise him/her to come back after 2 days if fever persists.

YOU HAVE REACHED THE END OF THE ALMANACH ASSESSMENT FOR FEBRILE ILLNESSES

After completing this thorough assessment you may have one or several diagnoses for a child.
You will find the recommendations for treatment and additional measures for the management of childhood illnesses in pages 10 to 18.

ASSESSMENT OF NON FEBRILE CHILDHOOD ILLNESSES

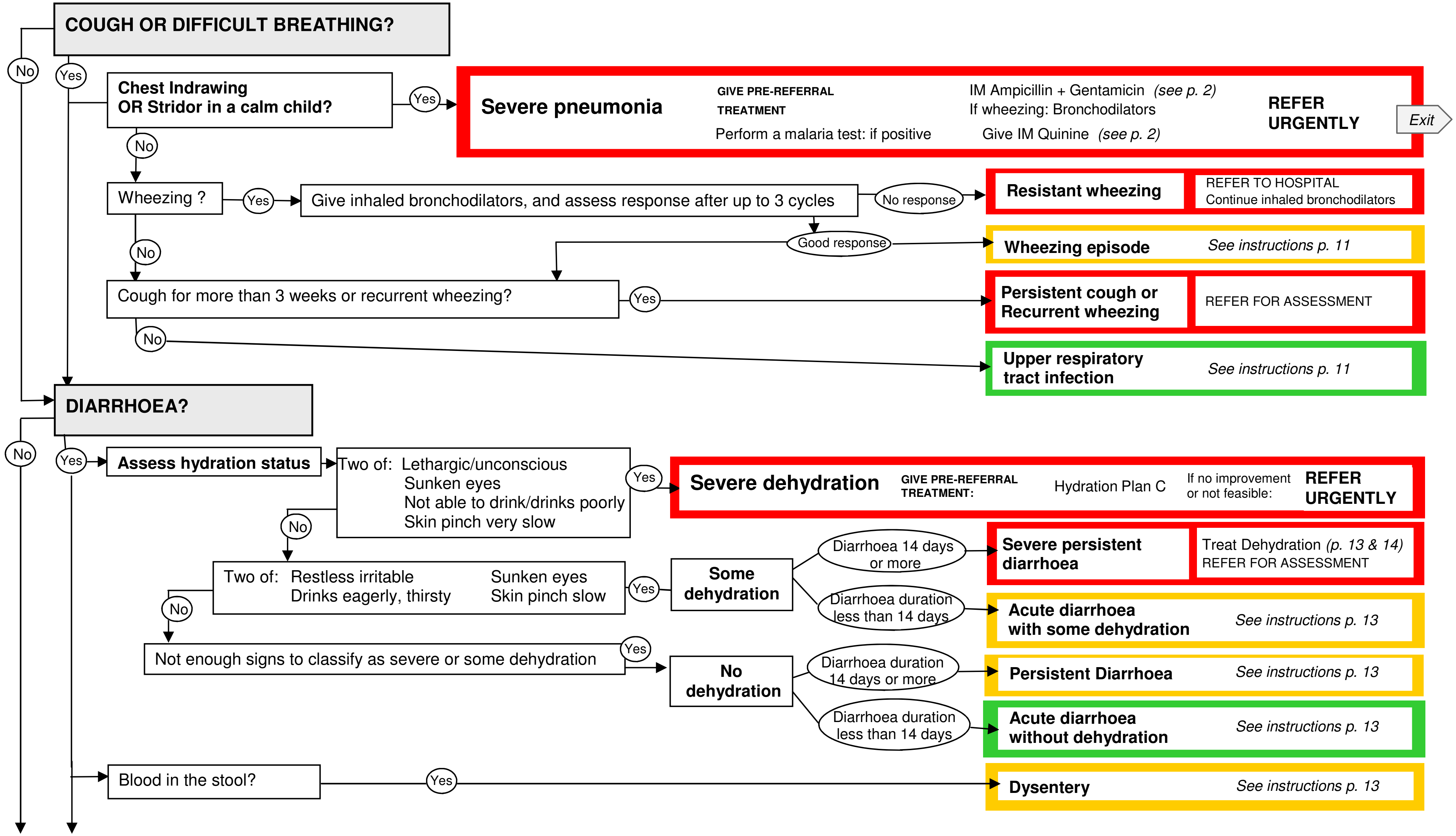

Complete assessment of non febrile illnesses p. 8

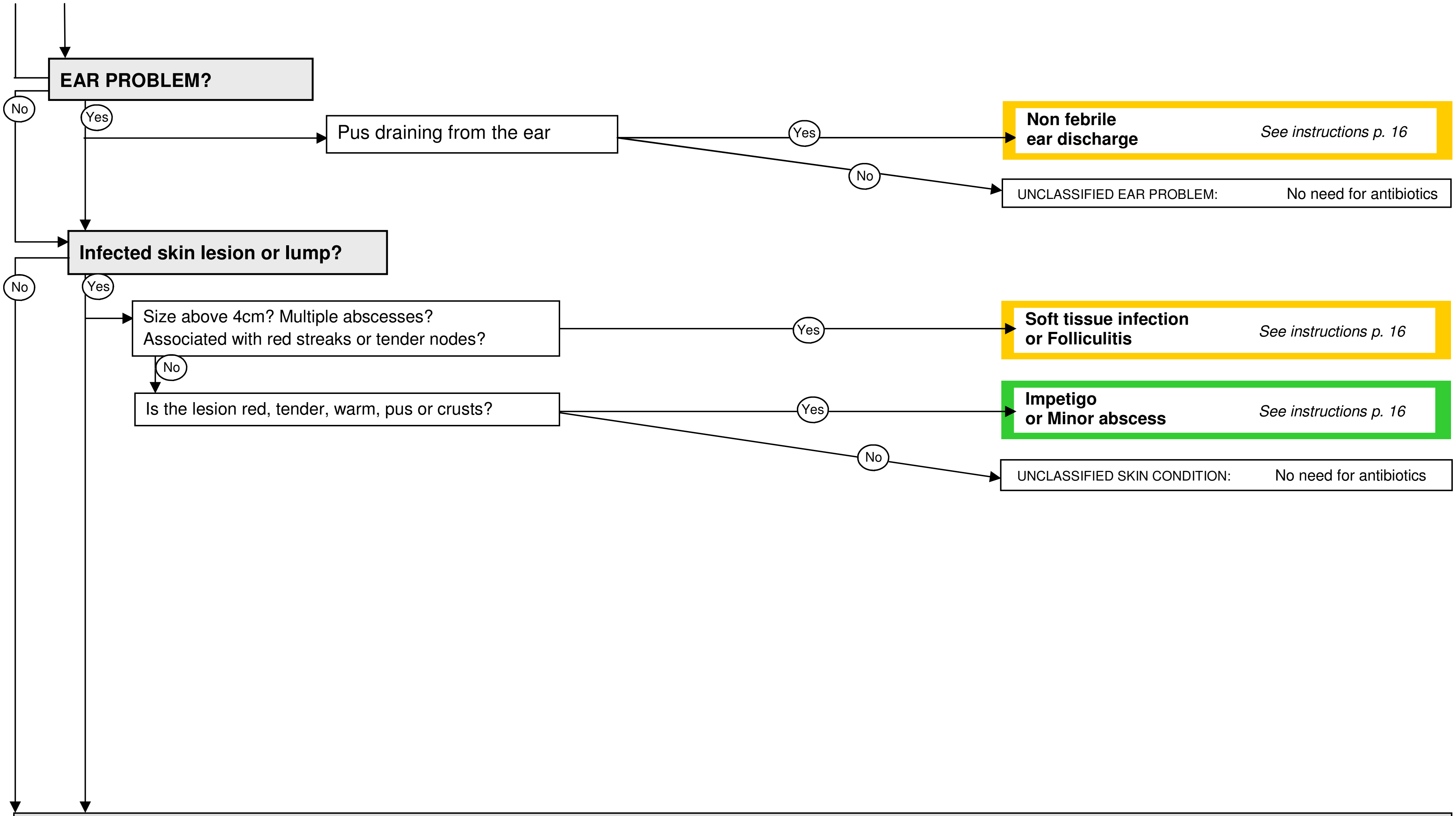

YOU HAVE REACHED THE END OF THE ALMANACH ASSESSMENT FOR NON FEBRILE ASSESSMENT

After completing this thorough assessment you may have one or several diagnoses for a child.
You will find recommendations for treatment and additional measures for the management of childhood illnesses in pages 10 to 18.

MANAGEMENT AND TREATMENT CHART

TABLE OF CONTENTS

General recommendations	……	10
Management of **COUGH** related diagnoses	Severe pneumonia, Persistent cough or Recurrent wheezing, Pneumonia,……	11
	Upper respiratory tract infection……	11
	Symptomatic treatment for Wheezing and Cough……	11
Management of **FEVER** related diagnoses	Persistent fever, Malaria, Urinary tract infection, Abdominal infection……	12
	Symptomatic treatment for fever……	12
Management of **DIARRHOEA** related diagnoses	Severe dehydration, Severe persistent diarrhoea, Persistent diarrhoea, Dysentery, ……	13
	Acute diarrhoea with some dehydration, Acute diarrhoea with no dehydration……	13
	Hydration plan A, B and C……	14
Management of **EAR** related diagnoses	Mastoiditis, Acute febrile ear discharge, Non febrile ear discharge, Chronic ear discharge, ……	15
	Acute ear infection……	15
	Clear the ear by dry wicking……	15
Management of **ANAEMIA** related diagnoses	……	15
Management of **MEASLES** related diagnoses	Severe Measles, Measles with mouth or eye complication, Measles……	16
Management of **SKIN** related diagnoses	Severe soft tissue infection, Soft tissue infection, Impetigo or minor abscess……	16
COMPLEMENTARY DOCUMENT	Use of a spacer……	17
	Weight-for-age charts……	17

GENERAL RECOMMENDATIONS

Always tell the caretakers the reason for giving the drug, and teach them how to give it at home if needed.
Demonstrate how to prepare a dose, watch them preparing the first dose, explain carefully how to give the drug and to complete the treatment even if the child gets better. Check the caretakers' understanding before they leave.

REASSURING THE CARETAKER when the RDT is negative

Reassure the caretaker that:

- the child does NOT have malaria
- the diagnostic test (RDT) is very accurate
- antimalarials will NOT help the child and you do not want to unnecessarily expose the child to side effects that may accompany these medications

MANAGEMENT AND TREATMENT CHART FOR **COUGH** RELATED DIAGNOSES

Severe pneumonia or Very severe disease
Give IM Ampicillin and Gentamicin *(see page 2 for instructions)*
Give inhaled bronchodilators if wheezing *(see below)*
REFER URGENTLY

Resistant wheezing
Continue inhaled bronchodilators, using a spacer, on the way to hospital *(see below)*
REFER URGENTLY

Persistent cough or recurrent wheezing
Refer to hospital for further assessment for Tuberculosis or Asthma

Pneumonia
Give Amoxicillin 25mg/kg, 2 times daily, for 5 days
Discuss HIV infection

Advise caretaker to:
Come back immediately if the child is not able to drink or breastfeed, or becomes sicker
Come back after 2 days if fever or difficult breathing persist

**Amoxicillin** 25mg/kg	CAPSULES 250 mg	SYRUP 125 mg/5 ml
4 - <6 kg (2 months to <4 months)		5 ml
6 - <14 kg (4 months to <3 years)	1	10 ml
14 - 19 kg (3 years to <5 years)	2	15 ml

Wheezing episode
Treat the wheezing in the clinic following the symptomatic treatment instructions below. If the child has a good response to the treatment, and doesn't need referral, continue the treatment at home:
At home: continue treatment with inhaled salbutamol, 3 to 4 times a day, for 5 days.
If inhaler not available: Use oral salbutamol (2 months up to 12 months: 1mg, 3 times daily; 2 months up to 4 years: 2mg, 3 to 4 times daily)

Advise caretaker to:
Come back immediately if the child is not able to drink/breastfeed, becomes sicker, or develops fever
Come back if the wheezing/difficult breathing persists after treatment

Upper respiratory tract infection (URTI) Explain the mother that the URTI is a viral disease that is self limiting

Advise caretaker to:
Relieve cough and soothe the throat with breast milk for an infant breastfed, or with tea with lemon or tea with honey for an older child
Come back immediately if the child is not able to drink or breastfeed, becomes sicker, develops fever, or develops fast/difficult breathing or wheeze
Come back after 5 days if the symptoms persist

ADDITIONAL SYMPTOMATIC TREATMENTS

Wheezing: *In the clinic:* Give inhaled bronchodilators: Salbutamol, using a spacer *(See page 17)*: From salbutamol metered dose inhaler (100 µg/puff) give 2 puffs. Reassess the child after 15 minutes. Repeat up to 3 times every 15 minutes before classifying pneumonia.

Cough and/or sore throat: To relieve cough and soothe the throat recommend the caretaker to use the safe remedies below:
For an infant who is exclusively breastfed: breast milk
For other children: breast milk, tea with honey, tea with lemon.

MANAGEMENT AND TREATMENT CHART FOR **FEVER** RELATED DIAGNOSES

Persistent fever — Refer to hospital for further assessment

Malaria

Give ALu:
- Give first dose in the clinic and observe the child for one hour. If the child vomits within an hour repeat the dose.
- Tell the mother to give second dose after 8 hours and then 2 times daily for further 2 days as shown in the table.
- ALu should be given with food.

Give one dose of paracetamol in the clinic for high fever (38.5 and above).

Advise caretaker to:
Come back immediately if the child is not able to drink or breastfeed, or becomes sicker
Come back after 2 days if fever persists
Always use insecticide treated mosquito net

**ALu** (artemether + lumefantrine)	Tablet *(artemether 20mg + lumefantrine 120mg)*					
	0h	8h	24h	36h	48h	60h
5* <15 kg (2 months to <3 years)	1	1	1	1	1	1
15 - <25 kg (3 years to <5 years)	2	2	2	2	2	2

**Children weighing less than 5 kg should be treated with Quinine Give Quinine tabs 300mg: 1/4, 3 times daily, for 7 days.*

Urinary tract infection (UTI) — Give Ciprofloxacin 15mg/kg, 2 times a day, for 5 days

Advise caretaker to:
Increase fluids
Come back immediately if the child is not able to drink/breastfeed, or becomes sicker
Come back after 2 days if fever persists

Possible intestinal bacterial disease — Give Ciprofloxacin 15mg/kg, 2 times a day, for 5 days

Advise caretaker to:
Increase fluids
Come back immediately if the child is not able to drink/breastfeed, or becomes sicker
Come back after 2 days if fever persists

**Ciprofloxacin** 15mg/kg	Tablet 250 mg	Tablet 500mg
<6 kg (less than 6 months)	1/4	
6- <14 kg (6 months to <3 years)	1/2	1/4
14-19 kg (3 years to <5 years)	1	1/2

SYMPTOMATIC MANAGEMENT AND TREATMENT FOR **FEVER**

For all children with FEVER: Give first dose of paracetamol in the clinic if high fever (38.5℃ and above)

Explain caretaker to:
Expose the child (decrease or remove the clothes)
Increase the fluids intake
Give paracetamol, 15mg/kg, every 6 hours until high fever or pain is gone

**Paracetamol** 15mg/kg	Syrup 120mg/5ml	Tablet 500mg
<4kgs	2ml	-
4-<10 kg (2 months to <1 year)	5ml	1/4
10-19 kg (1 year to <5 years)	-	1/2

MANAGEMENT AND TREATMENT CHART FOR **DIARRHOEA** RELATED DIAGNOSES

Severe dehydration
Give Hydration plan C *(see page 14)*
REFER URGENTLY to hospital if Plan C is not available in your health facility or if the child does not improve after 3 hours of treatment

Severe persistent diarrhoea
Give hydration plan B *(see page 14)*
and refer to hospital for further assessment

Acute diarrhoea with some dehydration
Give hydration plan B *(see page 14)*
Give zinc*

Advise caretaker to:
Continue feeding and give zinc*
Come back immediately if the child is not able to drink/breastfeed, becomes sicker, develops fever, or blood in stool
Come back after 5 days if diarrhoea persists

Persistent Diarrhoea
Give zinc* , Vitamin A treatment *(see page 16),* and multivitamin
Discuss HIV infection

Advise caretaker on feeding:
Continue breastfeeding, and give more frequent, longer breastfeeds, day and night
If the child takes other milk: Replace with increased breastfeeding OR Replace with fermented milk products, such as yoghurt OR Replace half the milk with nutrient—rich semisolid food

Advise caretaker to come back:
Immediately if the child is not able to drink/breastfeed, becomes sicker, develops fever, or blood in stool
After 5 days if diarrhoea persists

Dysentery
Give Ciprofloxacin 15mg/kg, 2 times a day, for 3 days
Give hydration according to dehydration status and continue feeding

Advise caretaker to come back:
Immediately if the child is not able to drink/breastfeed, becomes sicker, or develops fever
After 2 days if the diarrhoea persists

**Ciprofloxacin** 15mg/kg	Tablet 250 mg	Tablet 500mg
<6 kg (less than 6 months)	1/4	
6- <14 kg (6 months to <3 years)	1/2	1/4
14-19 kg (3 years to <5 years)	1	1/2

Acute diarrhoea without dehydration
Give hydration plan A *(see page 14)*
Give zinc*

Advise caretaker to:
Continue feeding and give zinc*
Come back immediately if the child is not able to drink/breastfeed, becomes sicker, develops fever, or blood in stool
Come back after 5 days if diarrhoea persists

***Give Zinc for 14 days to all children with diarrhoea:**
From 2 months up to 6 months: 1/2 tablet daily
6 months or more: 1 tablet daily

Show caretaker how to give zinc supplements:
Infants: dissolve tablet in a small amount of expressed breast milk, ORS or clean water in a cup.
Older children: tablets can be chewed or dissolved in a small amount of water.

HYDRATION PLAN A

Give extra fluids (as much as the chid will take): Give more frequent, longer breastfeeds, and give ORS or clean water in addition to breast milk. If the child is not exclusively breastfed, also give food-based fluids (such as soup, rice water, and yoghurt drinks).

Give ORS at home especially when the child has been treated with Plan B or Plan C during this visit OR when the child cannot return to a clinic if the diarrhoea gets worse.

Teach the caretaker how to mix and give ORS: Give 2 packets of ORS. Show how much fluid to give in addition to the usual fluid intake.
Tell her/him to give after each loose stool: 50 to 100ml for a child below 2 years of age, and 100 to 200ml for a child older than 2 years.
Give frequent sips from a cup. If the child vomits, wait 10 minutes. Then continue but more slowly. Continue giving extra fluid until the diarrhoea stops.

HYDRATION PLAN B Give ORS at the clinic over a 4 hours period.

Determine recommended amount of ORS to give over a 4 hours period using the table aside. If the child wants more ORS than shown, give more.

WEIGHT	< 6 kg	6 - <10 kg	10 - <12 kg	12 - 19 kg
AGE	<4 months	4 months to <12 months	12 months to <2 years	2 years to <5 years
ORS in ml	200 - 450	450 - 800	800 - 960	960 - 1600

Use the child's age only when you do not know the weight.

Show caretaker how to give ORS solution**:** Give frequent small sips from a cup. If the child vomits, wait 10 minutes. Then continue, but more slowly.
Continue breastfeeding whenever the child wants.
After 4 hours: Reassess the child and classify the child for dehydration. Select the appropriate plan to continue treatment. Begin feeding the child in clinic.
Before the caretaker leaves the clinic give her/him all explanation on how to continue the treatment at home *(see plan A).*

HYDRATION PLAN C: TREAT SEVERE DEHYDRATION QUICKLY: Follow the arrows. If answer is 'YES' go across. If 'NO' go down.

Can you give intravenous (IV) fluid immediately?

Yes →

Start IV fluid immediately. If the child can drink, give ORS by mouth while the drip is set up. Give 100 ml/kg Ringer's Lactate Solution (or, if not available, normal saline), divided as in table aside.

**AGE**	**First give 30 ml/kg in:**	**Then give 70 ml/kg in:**
Infants (under 12 months)	1 hour*	5 hours
Children (12 months up to 5 years)	30 minutes*	2 1/2 hours

*Repeat once if radial pulse is still very weak or not detectable. **Reassess the child every 1-2 hours.** If hydration status is not improving, give the IV drip more rapidly. Also give ORS (about 5 ml/kg/hour) as soon as the child can drink: usually after 3-4 hours (infants) or 1-2 hours (children).
Reassess an infant after 6 hours and a child after 3 hours. Classify dehydration. Then choose the appropriate plan (A, B, or C) to continue treatment.

No ↓

Is IV treatment available nearby (within 30 minutes)?

Yes →

Refer URGENTLY to hospital for IV treatment.
If the child can drink, provide the mother with ORS solution and show her how to give frequent sips during the trip or give ORS by naso-gastric tube.

No ↓

Are you trained to use a naso-gastric (NG) tube for rehydration?

No ↓

Can the child drink?

Yes →

Start rehydration by tube (or mouth) with ORS solution: give 20 ml/kg/hour for 6 hours (total of 120 ml/kg).

Reassess the child every 1-2 hours while waiting for transfer: If there is repeated vomiting or increasing abdominal distension, give the fluid more slowly. If hydration status is not improving after 3 hours, send the child for IV therapy.

After 6 hours, reassess the child. Classify dehydration. Then choose the appropriate plan (A, B or C) to continue treatment.

No ↓

REFER URGENTLY TO HOSPITAL for IV or NG treatment

NOTE:
If the child is not referred to hospital, observe at least 6 hours after rehydration to be sure mother can maintain hydration giving the child ORS solution by mouth.

MANAGEMENT AND TREATMENT CHART FOR **EAR** RELATED DIAGNOSES

Mastoiditis

Give IM Ampicillin and Gentamicin *(see page 2 for instructions)*
Give paracetamol for pain *(see p10)*

REFER URGENTLY

Acute febrile ear discharge

Give Amoxicillin 25mg/kg, 2 times daily, for 5 days
Dry the ear by wicking*
Discuss HIV infection

Advise caretaker to come back: immediately if the child is not able to drink or breastfeed, or becomes sicker
after 5 days if ear discharge persists

**Amoxicillin** 25mg/kg	CAPSULES 250 mg	SYRUP 125 mg/5 ml
4 - <6 kg (2 months to <4 months)		5 ml
6 - <14 kg (4 months to <3 years)	1	10 ml
14 - 19 kg (3 years to <5 years)	2	15 ml

Chronic ear discharge OR Non febrile ear discharge

Dry the ear by wicking* and instil quinolones eardrops just after wicking, 3 times daily, for 2 weeks.
(Quinolones eardrops may contain ciprofloxacin, norfloxacin or ofloxacin)
Discuss HIV infection

Advise caretaker to come back: immediately if the child is not able to drink or breastfeed, becomes sicker, or develops fever
after 5 days if ear discharge persists

Acute ear infection

Give paracetamol for pain *(see page 10)*

Advise caretaker to come back: immediately if the child is not able to drink or breastfeed, or becomes sicker
after 3 days if pain or fever persist

***Dry wicking to clear the ear of children with ear discharge**

Dry the ear at least 3 times daily:
Wash hands.
Roll a clean absorbent cloth or soft, strong tissue paper into a wick.
Place the wick in the child's ear. Remove the wick when wet.
Replace the wick with a clean one and repeat this steps until the ear is dry.

MANAGEMENT AND TREATMENT CHART FOR **ANAEMIA**

Anaemia Give Iron*, 1 dose daily, for a total of 2 months
Give Mebendazole 500mg single dose, if the child is one year or older and has not had a dose in the previous 6 months.

Check Malaria: Perform a Rapid diagnostic test (RDT) and treat if positive *(see page 11)*

Advise caretaker to come back: immediately if the child is not able to drink, becomes sicker, or develops fast breathing or fever
after 14 days for re-assessment

**IRON**	Ferrous sulfate 200 mg + 250 µg Folate (60 mg elemental iron)	Ferrous fumarate 100 mg per 5 ml (20 mg elemental iron per ml)
4 - <6 kg (2 to <4 months)		1.00 ml (< 1/4 tsp.)
6 - <10 kg (4 to <12 months)		1.25 ml (1/4 tsp.)
10 - <14 kg (1 to <3 years)	1/2 tablet	2.00 ml (<1/2 tsp.)
14 - 19 kg (3 to <5 years)	1/2 tablet	2.5 ml (1/2 tsp.)

MANAGEMENT AND TREATMENT CHART FOR **MEASLES** RELATED DIAGNOSES

Severe complicated measles

Give IM Ampicillin and Gentamicin (*see page 2 for instructions)*
Give Vitamin A and Tetracycline eye ointment *(see below)*

REFER URGENTLY

Measles with eye or mouth complication

Give vitamin A treatment *(See below)* to children from 6 months up 5 years, except if the child has had a dose in the past month.
Give paracetamol for pain relief.

Treat eye infection with tetracyclin eye ointment in both eyes, 4 times daily*: Clean both eyes: Wash hands, use clean cloth and water to gently wipe the pus. Then apply tetracyclin eye ointment: Squirt a small amount of ointment on the inside of the lower lid. Wash hands again. Treat until there is no pus discharge. Do not put anything else in the eyes.

Treat for mouth ulcers with Gentian Violet (GV) twice daily*: Wash hands. Wash the child's mouth with clean soft cloth wrapped around the finger and wet with salt water. Paint the mouth with half-strength gentian violet (0.25% dilution). Wash hands again. Continue using gentian violet for 48 hours after the ulcers have been cured.

Advise caretaker to come back: Immediately if the child is not able to drink/breastfeed, becomes sicker, develops fever
After 2 days if symptoms persist

Measles

Give Vitamin A treatment: Give a dose of Vitamin A to children from 6 months up to 5 years, except if the child has had a dose in the past month.

**AGE**	6 to <12 months	One year and older
**Vitamin A** dose	100 000 IU	200 000 IU

MANAGEMENT AND TREATMENT CHART FOR **SKIN** RELATED DIAGNOSES

Severe soft tissue or muscles infection

Give first dose of oral Cloxacillin (*see below)*

REFER TO HOSPITAL

Soft tissue infection or folliculitis

Give Cloxacillin, 25mg/kg, 4 times a day, for 5 days
Clean sores with antiseptic. Drain pus if fluctuance.

Advise caretaker to come back: After 1 day if symptoms persist
Immediately if the child is not able to drink, or becomes sicker.

**Cloxacillin** 25mg/kg	Syrup 125mg/5ml	Capsule 250 mg
4 - <6 kg (2 to <4 months)	5ml	
6 - <14 kg (4 mths to <3 yrs)	10ml	1
14 - 19 kg (3 to <5 years)	15ml	2

Impetigo or minor abscess

Clean sores with antiseptic. Drain pus if fluctuance.

Advise caretaker to come back: Immediately if the child is not able to drink/breastfeed, becomes sicker, develops fever
After 2 days if symptoms persist

* For local treatments: Explain to the caretaker what the treatment is and how it should be given. Tell her/him how often to do the treatment at home. If needed, give caretaker the tube of tetracycline ointment or a small bottle of gentian violet.

USE OF A SPACER
A spacer is a way of delivering the bronchodilator drugs effectively into the lungs. NO CHILD UNDER 5 YEARS OF AGE SHOULD RECEIVE AN INHALER WITHOUT A SPACER. A spacer works as well as a nebuliser if correctly used.

Spacers can be made in the following way:

Use a 500ml drink bottle or similar. Cut a hole in the bottle base in the same shape as the mouthpiece of the inhaler, with a sharp knife. Cut the bottle between the upper quarter and the lower 3/4 and disregard the upper quarter of the bottle.

Cut a small V in the border of the large open part of the bottle to fit to the child's nose and be used as a mask. Flame the edge of the cut bottle with a candle or a lighter to soften it. In a small baby, a mask can be made by making a similar hole in a plastic (not polystyrene) cup. Alternatively commercial spacers can be used if available.

To use an inhaler with a spacer*:

Remove the inhaler cap. Shake the inhaler well. Insert mouthpiece of the inhaler through the hole in the bottle or plastic cup. The child should put the opening of the bottle into his mouth and breath in and out through the mouth. A carer then presses down the inhaler and sprays into the bottle while the child continues to breath normally. Wait for three to four breaths and repeat. For younger children place the cup over the child's mouth and use as a spacer in the same way.

** If a spacer is being used for the first time, it should be primed by 4-5 extra puffs from the inhaler.*

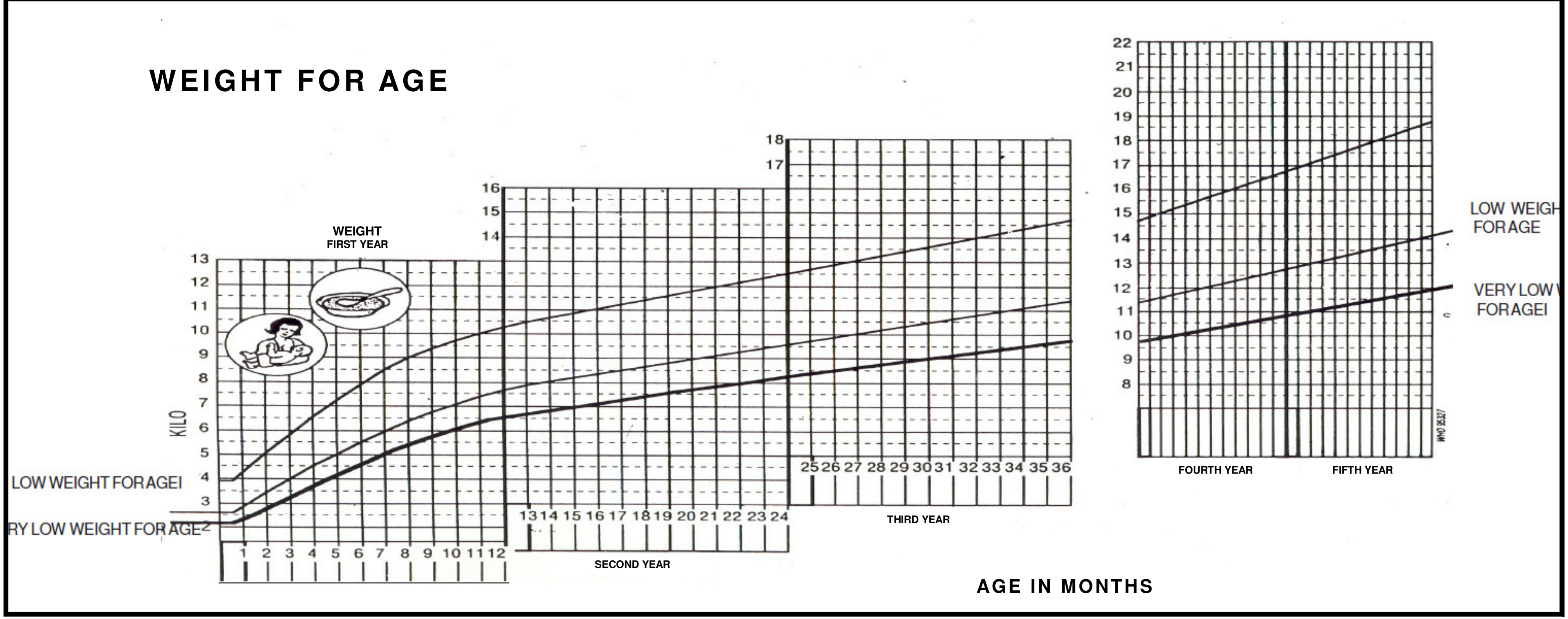

Supplement: S1 Fig — (PDF) [file pone.0127674.s001.pdf]
